# A comparison of melatonin and α-lipoic acid in the induction of antioxidant defences in L6 rat skeletal muscle cells

**DOI:** 10.1007/s11357-015-9824-7

**Published:** 2015-08-07

**Authors:** Gaia Favero, Luigi Fabrizio Rodella, Lorenzo Nardo, Lorena Giugno, Marco Angelo Cocchi, Elisa Borsani, Russel J. Reiter, Rita Rezzani

**Affiliations:** 1 Division of Anatomy and Physiopathology, Department of Clinical and Experimental Sciences, University of Brescia, Viale Europa 11, 25123 Brescia, Italy; 2 University of Brescia, Brescia, Italy; 3 Department of Radiology and Biomedical Imaging, University of California San Francisco, 185 Berry Street, Suite 350, San Francisco, CA 94107 USA; 4 Department of Cellular and Structural Biology, University of Texas Health Science Center, San Antonio, TX 78229 USA

**Keywords:** Melatonin, α-Lipoic acid, Hydrogen peroxide, Oxidative stress, Skeletal muscle cell

## Abstract

Aging is characterized by a progressive deterioration in physiological functions and metabolic processes. The loss of cells during aging in vital tissues and organs is related to several factors including oxidative stress and inflammation. Skeletal muscle degeneration is common in elderly people; in fact, this tissue is particularly vulnerable to oxidative stress since it requires large amounts of oxygen, and thus, oxidative damage is abundant and accumulates with increasing age. Melatonin (*N*-acetyl-5-methoxytryptamine) is a highly efficient scavenger of reactive oxygen species and it also exhibits beneficial anti-inflammatory and anti-aging effects. This study investigated the susceptibility of rat L6 skeletal muscle cells to an induced oxidative stress following their exposure to hydrogen peroxide (50 μM) and evaluating the potential protective effects of pre-treatment with melatonin (10 nM) compared to the known beneficial effect of alpha-lipoic acid (300 μM). Hydrogen peroxide-induced obvious oxidative stress; it increased the expression of tumour necrosis factor-alpha and in turn promoted nuclear factor kappa-B and overrode the endogenous defence mechanisms. Conversely, pre-treatment of the hydrogen peroxide-exposed cells to melatonin or alpha-lipoic acid increased endogenous antioxidant enzymes, including superoxide dismutase-2 and heme oxygenase-1; moreover, they ameliorated significantly oxidative stress damage and partially reduced alterations in the muscle cells, which are typical of aging. In conclusion, melatonin was equally effective as alpha-lipoic acid; it exhibited marked antioxidant and anti-aging effects at the level of skeletal muscle in vitro even when it was given in a much lower dose than alpha-lipoic acid.

## Introduction

Since individuals in Western societies are living longer, there is an increasing necessity to understand the pathophysiological processes of aging. Aging is associated with the progressive accumulation of oxidative debris, which contributes to functional inefficiency of cells, thereby inducing additional free radical production and metabolic inefficiency. Thus, aging becomes a vicious cycle in which molecular processes fail resulting in accumulated oxidative damage causing physiological degeneration; this further exaggerates the production of free radicals (Cencioni et al. 2013).

A decline in skeletal muscle mass and function is a consequence of aging as well as of pathological processes including cancer, myopathies, and sepsis (Garatachea and Lucía 2013; Park et al. 2013). Muscle degeneration is characterized by the deregulation of calcium homeostasis, enhanced protease activity, loss of cytoskeletal and sarcolemmal integrity, impaired energy metabolism and oxidative stress, all of which contribute to muscle atrophy and degeneration (Hopf et al. 2007; Park et al. 2013).

Oxidative stress is a situation related to a serious imbalance between the production of oxidants, such as hydrogen peroxide (H_2_O_2_) and superoxide anion (O_2_^·−^), and antioxidant defence mechanisms culminating in cellular apoptosis or necrosis. The loss of cells due to programmed cell death is a consequence of/or contributes to many diseases as well as age-related deterioration (Halliwell 2003; Reiter 1995; Vinayaga et al. 2006). Skeletal muscle is particularly vulnerable to oxidative stress because it requires a large quantity of oxygen, and thus, muscle cells accumulate significant amounts of oxidative damage over time. Reactive oxygen species (ROS) damage many cellular components including DNA, lipid membranes and proteins (Aoi et al. 2004; Kozlovsky et al. 1997). Fortunately, cells also possess a variety of antioxidant defence systems. These include small molecules such as α-tocopherol and ascorbic acid that intervene as sacrificial molecules in redox cycles (Harris 1992). Antioxidants also include specific inducible antioxidant enzymes such as superoxide dismutase (SOD), catalase, heme oxygenase (HO) and glutathione peroxidase which either convert oxygen radicals into less offensive products or prevent their formation (Devasagayam et al. 2004; Kozlovsky et al. 1997). As signalling molecules, ROS can activate numerous cellular stress-sensitive pathways, such as the phosphorylation of c-Jun N-terminal kinase (JNK) and nuclear factor kappa-B (NF-kB) (Min et al. 2009), which cause cell damage. Also, protein kinase signalling pathways are activated (Sebastián et al. 2012). Tumour necrosis factor-α (TNF-α), a cytokine produced by monocytes and macrophages (Kozlovsky et al. 1997; Sprague and Khalil 2009), is a rapid and potent activator of NF-kB (Li 2003; Park et al. 2013; Sen et al. 1997). NF-kB, as a transcription factor, stimulates the expression of a number of genes related to oxidative stress, immune responses, inflammation and apoptosis. It is regulated in a complex manner by the ubiquitin-proteasome system; degradation of the NF-kB inhibitor, IkK, by the proteasome results in activation of NF-kB (Perkins 2007; Skaug et al. 2009; Vriend and Reiter 2014).

Melatonin is a small, highly conserved pineal secretory product with numerous receptor-mediated and receptor-independent actions (Reiter et al. 2007). Melatonin is perhaps best known for its mediation of circannual regulation of metabolism and reproductive competence in photosensitive species and its ability to influence circadian processes that are ubiquitous in organisms and in cells (Campos Costa et al. 2013). However, it is also well known for its ability to neutralize free radicals, reduce inflammation and defer age-related dysfunction of several organs (Favero et al. 2014; Galano et al. 2015; Hardeland 2013; Reiter et al. 2014).

Alpha-lipoic acid (1,2-dithiolane-3-pentanoic acid—LA) is a powerful antioxidant which might act by three distinct actions: (i) reactive oxygen species-scavenging activity; (ii) capacity to regenerate endogenous antioxidants, such as glutathione and vitamins C and E and (iii) metal-chelating activity (Arroll et al. 2014; Packer et al. 1995).

There are rather few studies that have compared the potential beneficial actions of melatonin with those of alpha-lipoic acid against induced atrophy and oxidative stress in a well-characterized in vitro model of skeletal muscle, rat L6 skeletal muscle cells (Dott et al. 2014; Jaiswal et al., 2015; Rachek et al. 2007). Herein, we first analysed the effects of oxidative stress induced by H_2_O_2_ on L6 myotubes; thereafter, we evaluated the protective effects of pre-treatment with melatonin and compared its antioxidant activity with the known beneficial effects of LA on L6 cells (Maddux et al. 2001).

## Material and methods

L6 rat skeletal myoblasts, obtained from the Experimental Zooprophylactic Institute of Lombardy and Emilia Romagna “Bruno Ubertini”, were cultured in Dulbecco’s modified Eagle’s medium (DMEM) supplemented with 10 % heat-inactivated foetal bovine serum (FBS), penicillin (100 U/ml) and streptomycin (100 μg/ml) and incubated at 37 °C in a humidified atmosphere with 5 % carbon dioxide and 95 % air atmosphere. The cells, grown in monolayer, were allowed to differentiate and propagate, and the growth medium was replaced with differentiation medium composed of DMEM supplemented with 2 % FBS, penicillin (100 U/ml) and streptomycin (100 μg/ml), as previously described by Park et al. (2013) and Vinayaga Moorthi et al. (2006). Reduction of serum allowed cell-to-cell fusion and formation of myotubes. In particular, the differentiation in myotubes was allowed to progress in 10–14 days and the medium was changed every 48 h before experimentation. Myogenic differentiation in myotubes was confirmed by morphological analyses using light microscopy, observing closely the alignment, elongation and the fusion of cells.

The L6 myoblasts were plated in culture six wells, induced to differentiate and randomly divided into the following experimental groups: control, control plus melatonin, control plus LA, hydrogen peroxide (H_2_O_2_) treatment, melatonin pre-treatment and then H_2_O_2_ incubation, and LA pre-treatment and then H_2_O_2_ incubation. The cells of each experimental group were treated at confluence; L6 myotubes reached confluence in about 1 week.

The cells treated with melatonin were incubated for 24 h in a differentiation medium supplemented with powder of pure melatonin (Melapure™ by Flamma S.p.A., Chignolo d’Isola, Italy) at a final concentration of 10 nM, the maximal effective concentration as determined in a previous study of Park et al. (2013), in which melatonin, in a dose-dependent manner, significantly restored cell survival and attenuated oxidative stress generation and muscle cell proteolysis. Whereas, the myotubes, treated with LA for 24 h, were added to powder of pure LA (Labochim S.p.A., Milan, Italy) at the final concentration of 300 μM, the maximal effective dose as determined by Maddux et al. (2001), which showed that LA protect L6 muscle cells from oxidative stress-induced insulin resistance in vitro. Melatonin and LA were directly added to the six-well culture plates, without changing the differentiation medium or washing, to avoid ROS production.

In other experimental groups, the myotubes were treated with H_2_O_2_ at the concentration of 50 μM for 1 h, as previously described by Blair et al. (1999). H_2_O_2_ is a toxic species that is widely used in cell studies to induce oxidative stress. It is often used as an exogenous oxidant treatment to promote free radical generation and proteolytic signalling pathways in both isolated skeletal muscles and in in vitro myotubes (Li et al. 2003; McClung et al. 2009). However, the concentration of H_2_O_2_ used in experiments differs widely in different cell types. In experiments conducted using myoblasts, a low concentration (1–10 μM) of H_2_O_2_ was found to enhance growth, while moderate and high concentrations (50–300 μM) were detrimental to the cells (Caporossi et al. 2003; Orzechowski et al. 2002; Shao et al. 2012).

### Cell viability assay

At the end of treatment, the myotubes were collected and resuspended in phosphate buffer solution (PBS), to which was added 0.4 % trypan blue. Counts of viable (unstained) and non-viable (blue-stained) cells were made using a light microscope with a haemocytometer, and the percentage of viable cells was calculated, as previously reported by Rezzani et al. (2014). The assessment of cell viability was carried out by two independent observers blinded to cell treatments. In case of dispute concerning interpretation, the case was reconsidered until agreement was reached.

### Measurement of myotube diameter

At the end of treatment, five images were collected per experimental well, and from each image, the 10 largest myotubes were measured using an image analyser (Image Pro Plus; Milan, Italy). In particular, the myotube diameter, expressed in micrometer, was calculated by two independent observers blinded to the experimental group analysed. The myotube diameter was calculated from the average of three independent measurements per myotube.

### Immunofluorescence and morphometrical assay

At the end of the treatments, L6 cells of each experimental group were fixed in 4 % buffered paraformaldehyde for 10 min, washed in PBS and incubated in 0.3 % bovine serum albumin for 1 h at room temperature and then overnight at 4 °C with the following antibodies: TNF-α (dilution 1:300; Santa Cruz Biotechnology Inc., Dallas, TX, USA), NF-kB (dilution 1:300; Abcam, Cambridge, UK), superoxide dismutase 2 (SOD2) (dilution 1:300; Abcam, Cambridge, UK) and heme oxygenase-1 (HO-1) (dilution 1:300; Abcam, Cambridge, UK). Thereafter, the myotubes were labelled with the respective conjugated secondary antibody (anti-goat or anti-mouse Alexa Fluor 546 or anti-rabbit Alexa Fluor 488—diluted 1:200; Life Technologies, Grand Island, NY, USA). Finally, the cells were counterstained with 4′,6-diamidino-2-phenylindole (DAPI), mounted and observed with a confocal microscope (510 Meta Zeiss, Oberkochen, Germany), as previously described by Rodella et al. (2013). The control for the immunofluorescence was performed by omitting the primary antibody and in the presence of isotype-matched total immunoglobulin G.

Immunopositivity (staining intensity) of immunofluorescence analyses was computed by two independent observers blinded to the cell treatments using an optical fluorescent microscope equipped with an image analyser (Image Pro Plus; Milan, Italy). The immunopositivity was calculated by measuring, for each experimental group, 50 random fields with the same area (0.04 mm^2^), as previously described by Rezzani et al. (2014). The levels of immunopositivity are expressed as arbitrary units (AU). The data were pooled to calculate a mean value, and statistical significance of differences among the experimental groups was evaluated by ANOVA and Bonferroni test with significance set at *p* < 0.05.

### Tumour necrosis factor-alpha ELISA procedure

TNF-α concentration in cell lysates was measured using a commercial in vitro enzyme-linked immunosorbent assay (ELISA). In particular, we used a specific rat sandwich ELISA kit (Abcam, Cambridge, UK), according to manufacturer’s guidelines. The optical density was determined at 450 nm in a microplate reader, and the TNF-α levels were expressed as pg/mL.

## Results

### Myotube viability and atrophy

The incubation of L6 cells with H_2_O_2_ for 1 h reduced cell survival by 17.8 % (*p* < 0.05); the dead cells that underwent apoptosis appeared 5 to 10 μm in size. The majority of control myotubes, treated or not with melatonin, appeared normal in cells ranging from 10 to 18 μm in size. Interestingly, H_2_O_2_-induced inhibition of cell survival was significantly alleviated by melatonin pre-treatment, increasing cell survival by 6.7 % (*p* < 0.05) with respect to cells treated only with H_2_O_2_. Moreover, we observed that the treatment of L6 myotubes with LA caused the same trend in terms of survival as the melatonin-treated group; in particular, the pre-treatment of L6 cells with LA lowered cell death by 8.8 % (*p* < 0.05) relative to cell death induced by H_2_O_2_ only.

**Fig. 1:**
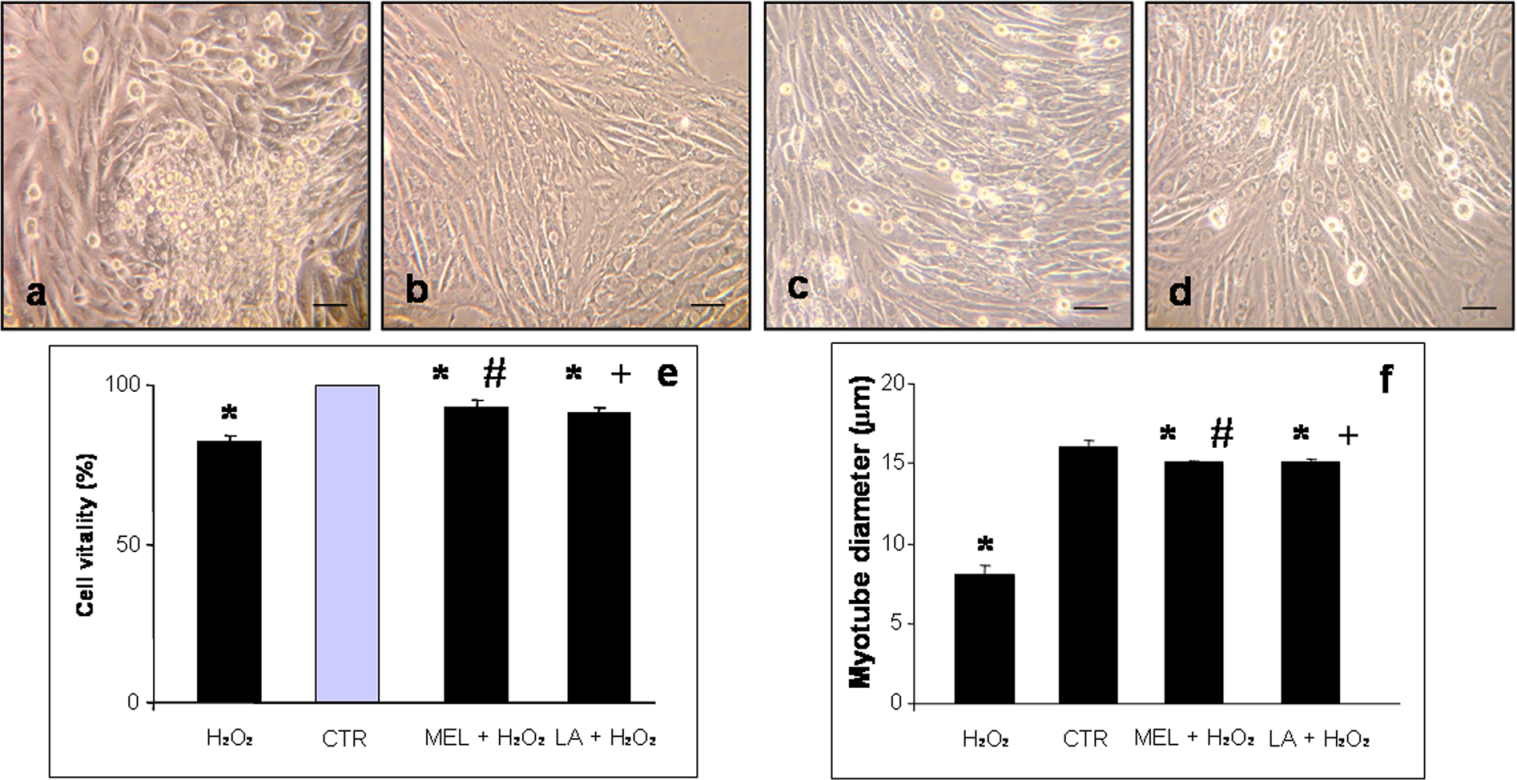
Photomicrographs of L6 myotubes incubated with hydrogen peroxide—H_2_O_2_ (**a**), control—CTR (**b**), pre-treated with melatonin and then incubated in hydrogen peroxide—MEL + H_2_O_2_ (**c**) or pre-treated with LA and then incubated in hydrogen peroxide—LA+ H_2_O_2_ (**d**). The myotube viability is expressed as percentage of viable cells (**e**) and cell size as myotube diameter in micrometer (**f**). * *p* < 0.05 vs control cells, #*p* < 0.05 vs hydrogen peroxide-treated cells and +*p* < 0.05 vs pre-treated with melatonin and then incubated in hydrogen peroxide

Myotube diameter was analysed as an indicator of skeletal muscle cell atrophy in response to H_2_O_2_ treatment. Myotube diameter decreased by 28.8 % (*p* < 0.05) in H_2_O_2_-incubated cells with respect to those in control cells, and pre-treatment with melatonin or LA increased the diameter by 24.4 and 23.2 % (*p* < 0.05), respectively (for more details see Fig. 1).

Interestingly, we observed that control cells treated or not with melatonin or LA for 24 h did not exhibit significant differences relative to cell vitality or morphological features; thus, we considered these as a single group defined generically as “control”.

### Oxidative stress and antioxidant defence

**Fig. 2:**
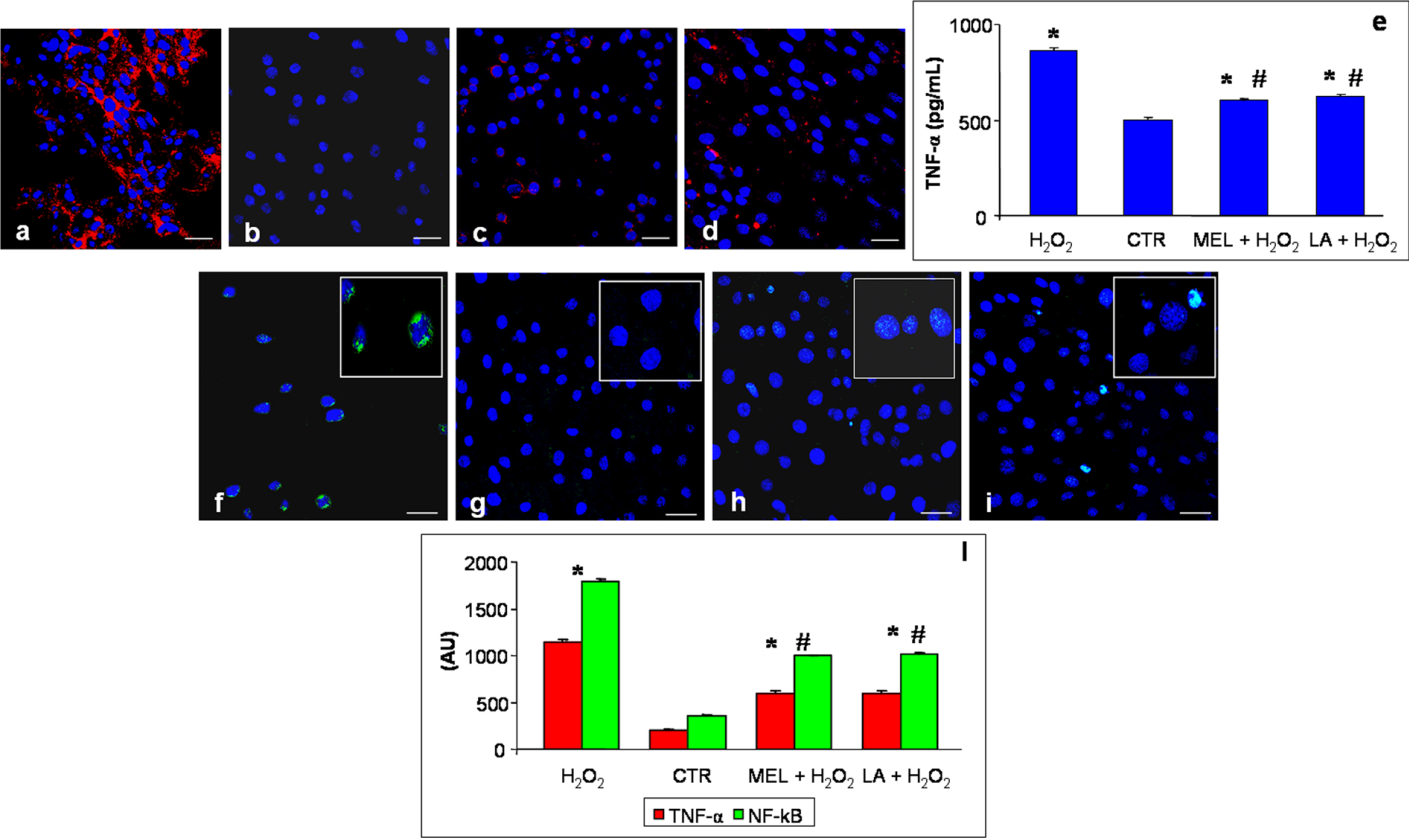
Immunofluorescence analyses of tumour necrosis factor-α—TNF-α (**a**–**d**) and nuclear factor kappa-B—NF-kB (**f**–**i**) in L6 myotubes incubated with hydrogen peroxide—H_2_O_2_ (**a**, **f**), control—CTR (**b**, **g**), pre-treated with melatonin and then incubated in hydrogen peroxide—MEL + H_2_O_2_ (**c**, **h**) and pre-treated with LA and then incubated in hydrogen peroxide—LA+ H_2_O_2_ (**d**, **i**). The *insets* show the NF-kB-positive nuclei of each experimental group. *Scale bar* = 20 μm. The graph (**e**) represents the quantitative measure of TNF-α in cell lysates (pg/mL) and summarizes the quantitative analysis of immunopositivities of both TNF-α and NF-kB (**i**). * *p* < 0.05 vs control cells and #*p* < 0.05 vs hydrogen peroxide-treated cells

With regard to TNF-α staining (red colour in figures), H_2_O_2_ myotubes showed a strong/very strong immunopositivity; in contrast, control cells exhibited absent/very weak immunostaining and the cells pre-treated with melatonin or LA both exhibited a weak expression (Fig. 2a–d). In particular, TNF-α staining exhibited a positive and diffuse staining at the cytoplasmic level with no positivity observed in the nucleus. Furthermore, these results are confirmed also by the quantitative measurement of TNF-α level in cell lysates (Fig. 2e).

NF-kB immunopositivity (green staining) was higher in H_2_O_2_-incubated cells compared to that in control myotubes; in particular, H_2_O_2_ incubation induced strong/very strong staining compared to an absent/very weak signal in control cells (Fig. 2f, g). The pre-treatment with melatonin or with LA reduced NF-kB immunopositivity significantly, showing, respectively, very weak and weak/moderate signals (Fig. 2h, i). Of interest, NF-kB immunostaining was evident in the nucleus of cells and no positivity or very weak signal was observed at the cytoplasmic level. All findings related to oxidative stress markers were also confirmed by quantitative analyses, as summarized in Fig. 2l.

**Fig. 3:**
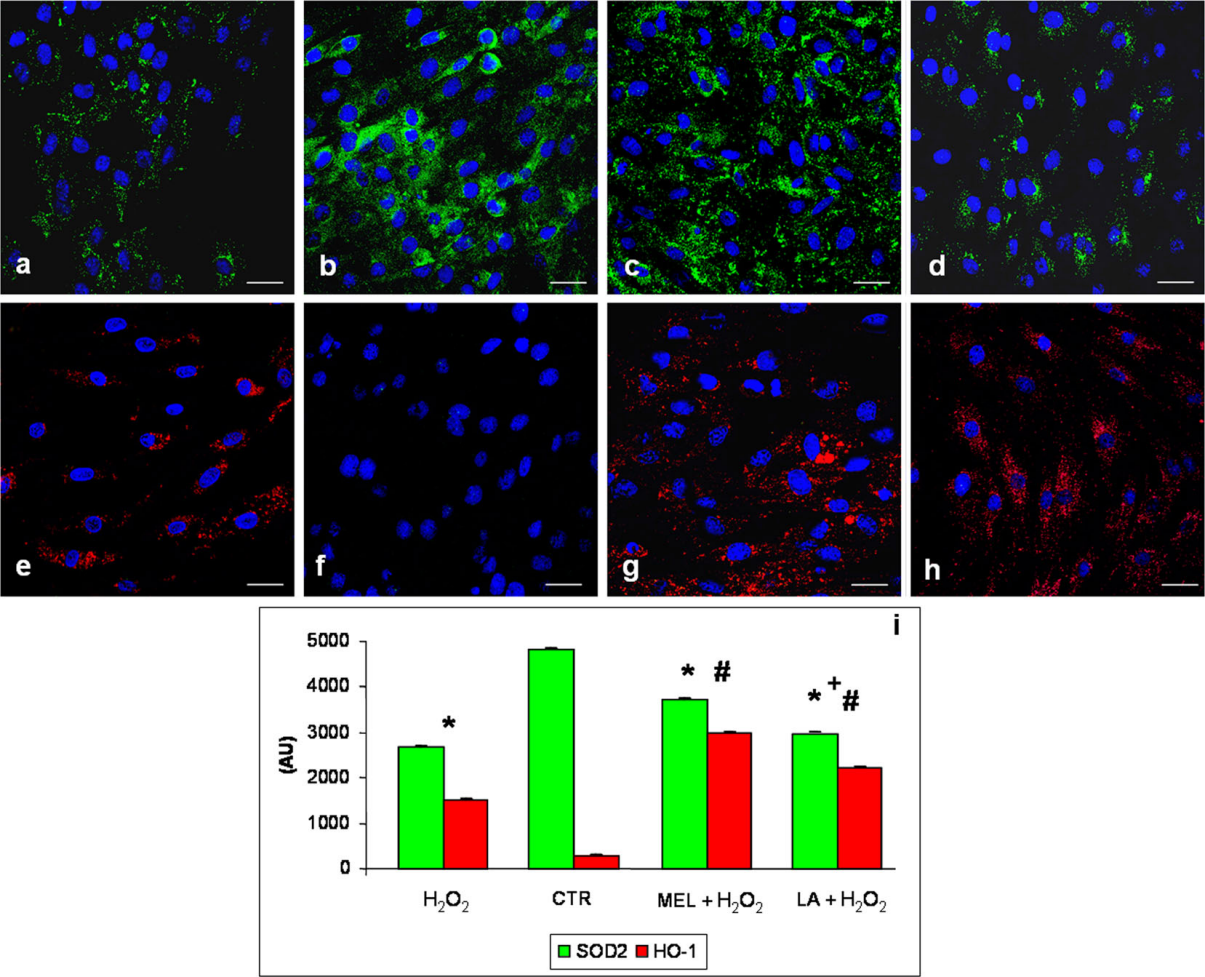
Immunofluorescence analyses for superoxide dismutase 2—SOD2 (**a**–**d**) and heme oxygenase-1—HO-1 (**e**–**h**) in L6 myotubes incubated with hydrogen peroxide—H_2_O_2_ (**a**, **e**), control—CTR (**b**, **f**), pre-treated with melatonin and then incubated in hydrogen peroxide—MEL + H_2_O_2_ (**c**, **g**) and pre-treated with LA and then incubated in hydrogen peroxide—LA+ H_2_O_2_ (**d**, **h**). Nuclei were stained with DAPI. *Scale bar* = 20 μm. The graph (**i**) summarizes the quantitative analysis of immunopositivities. * *p* < 0.05 vs control cells, #*p* < 0.05 vs H_2_O_2_-treated cells and +*p* < 0.05 vs pre-treated with melatonin and then incubated in hydrogen peroxide

The expression of SOD2 (green staining) was lower in H_2_O_2_-treated cells than in control myotubes, in which it was moderately expressed (Fig. 3a, b). However, both pre-treatment with melatonin or with LA increased SOD2 expression (Fig. 3c, d). In particular, H_2_O_2_ incubation induced a very weak expression compared to the pre-incubation period with melatonin or LA treatments causing, respectively, moderate and weak/moderate signalling. Specifically, the immunopositivity was evident in the cytoplasm as “granules”, suggesting mitochondrial SOD2 expression. No positivity was observed in the nucleus.

HO-1 immunostaining, identified in red, was weak in the L6 myotubes incubated in H_2_O_2_ relative to that in the control cells, in which there was no signal. Interestingly, pre-treatment with melatonin or LA increased significantly, with moderate/strong signals for HO-1 expression (Fig. 3e, h). HO-1 immunostaining was evident in the cytoplasm of the myotubes and there was no positivity at the nuclear level. These observations were confirmed by morphometrical analyses, as summarized in Fig. 3i.

## Discussion

Herein, we compared the effects of melatonin or LA on atrophy and oxidative stress of cultured rat L6 myotubes exposed to H_2_O_2_. In accordance with Blair et al. (1999), H_2_O_2_ was shown to be cytotoxic and induced growth retardation and cell death. H_2_O_2_ may also be an important molecule that regulates muscle growth; however, when its concentration is excessive or when the antioxidant defence is severely compromised, high levels of H_2_O_2_ cause pathology with consequent cell death via apoptosis and/or necrosis (Orzechowski et al. 2003). In our studies, it was shown that H_2_O_2_ did not induce mitogenicity, but it abrogated growth and induced oxidative stress, atrophy and apoptosis. Reduced mitogenicity in rat L6 muscle cells results from the oxidative/nitrosative stress developed in response to ROS donors (Orzechowski et al. 2002; 2005).

Previous studies have demonstrated that oxidants and TNF-α stimulate NF-kB translocation into the nucleus as well as the expression of genes related to oxidative stress, cytokines and adhesion molecules in L6 cells (Aoi et al. 2004; Reid and Li 2001; Vriend and Reiter 2014); this is consistent with the current results. In particular, the results of the present study indicate that H_2_O_2_-induced oxidative stress was increased and endogenous antioxidant mechanism reduced after TNF-α stimulation and NF-kB translocation into the nucleus. Pre-incubation with melatonin or LA limited the atrophic and oxidative stress responses, which supports the antioxidant and anti-aging properties of melatonin (Favero et al. 2014; Hardeland 2013; Reiter et al. 2014) and confirms the protective effect of LA against oxidative stress (Maddux et al. 2001).

Oxidative insults to cells are facilitated by the impairment in the function of endogenous antioxidant enzymes (Pandey and Rizvi 2011). In fact, the absence of a compensatory increase in endogenous antioxidant activity results in the activation of stress-sensitive signalling pathways (Dhanya et al. 2014). In this context, the concentration of H_2_O_2_ used in the current study was sufficient to induce cell death, resulting in a weak induction of endogenous antioxidants (SOD and HO-1) and a significant induction of oxidative stress and atrophy, as also reported by McClung et al. (2009) in C2C12 myotubes.

To reduce atrophy and oxidative stress, we pre-treated the cells with melatonin or LA and observed that H_2_O_2_-induced apoptogenic effects were attenuated. Melatonin, acting both as a direct and indirect antioxidant, ameliorates mitogenicity also increasing SOD2 and HO-1 expression which, in turn, affected protein synthesis as did H_2_O_2_. Also, LA reduced oxidative stress and increased endogenous antioxidants, but with a less obvious protective effect than melatonin.

Fischer et al. (2013) reported a melatonin-mediated enhancement of the endogenous antioxidative enzyme activity network as an important and protective mechanism against ultraviolet radiation-induced oxidative damage to the skin. Interestingly, they observed that melatonin was not only an effective direct radical scavenger but also functioned as an indirect antioxidant through enhancement of antioxidative enzyme expression and as a counteracting substance against oxidative stress-induced DNA damage; these multiple mechanisms of action render melatonin a highly potent antioxidant against oxidative-induced damage.

In this study, we demonstrated that HO-1 activity is stimulated by both H_2_O_2_ and especially by melatonin pre-treatment in L6 skeletal myotubes and that this activation is associated with a concomitant important reduction in oxidative stress. Our results support the finding of Essig et al. (1997) in which it was postulated that HO-1 is stimulated in rat skeletal muscle and mediates cell adaptation to oxidative stress during muscle contraction. HO-1 is a strong candidate as a key player in protecting against skeletal muscle damage and has been reported to perform this action in other cell types as well (Hirai et al. 2003; Lee et al. 2003; Vesely et al. 1998). The manner by which an increased activity of HO-1 can be cytoprotective is actually not known. A major function of HO-1 is to catabolize heme to generate bilirubin, carbon monoxide and free iron, all of which may play direct or indirect roles in the cytoprotective effects of HO-1 (McArdle et al. 2004; Morse and Choi 2002).

Because induction of ROS production appears to play a central function in several diseases associated with aging, antioxidant upregulation represents a fundamental mechanism by which melatonin may exert therapeutic effects against oxidative stress and atrophy associated with these conditions. The protective effects of melatonin and its metabolites are currently attributed to melatonin’s radical scavenging and antioxidant properties (Galano et al, 2013; Zhang and Zhang 2014). In this study, both direct and indirect antioxidant properties of melatonin were observed; this indoleamine directly scavenges a variety of the ROS including H_2_O_2_ and the highly deleterious hydroxyl radical (Reiter et al. 2002; Tengattini et al. 2008). Additionally, melatonin indirectly stimulates the expression and activity of antioxidant enzymes, including glutathione peroxidase, SOD, glutathione reductase and catalase as reported also by Park et al. (2013) and Rodriguez et al. (2004). In particular, Park et al. (2013) examined melatonin’s potential to protect skeletal muscle cells against TNF-α-induced muscle atrophy in an in vitro model and observed that it increased cellular antioxidant mechanisms.

**Fig. 4:**
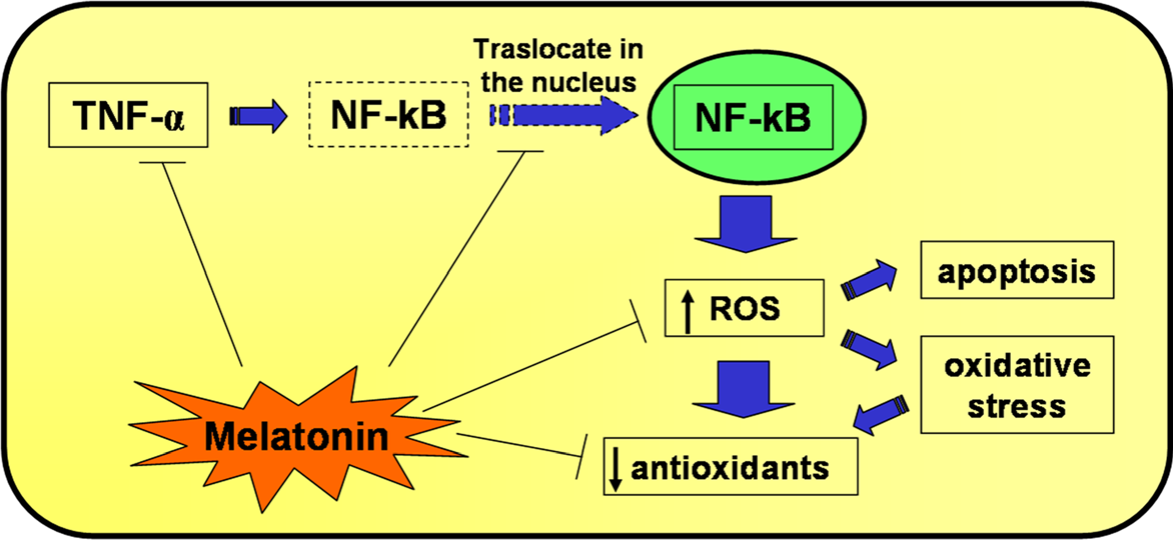
A schematic representation of hydrogen peroxide-mediated alterations in skeletal muscle cell and an illustration of the protective antioxidant and anti-aging effects of melatonin

Melatonin protects L6 myotubes from damage induced by H_2_O_2_ (Fig. 4); this effect might be achieved by maintaining the integrity of cellular integrity due to a reduction in oxidative stress and apoptosis and by increasing antioxidant defence responses thereby exerting a protective effect similar to that observed by Maddux et al. (2001) with LA pre-treatment. In the current study, we evaluated the protective actions of the pre-treatment with melatonin and compared its antioxidant activity with the known beneficial effects of LA. This comparison showed that melatonin pre-treatment was more efficient with respect to LA pre-treatment in increasing endogenous antioxidant enzymes (SOD2 and HO-1); this was especially obvious considering the respective concentrations of the molecules used, i.e., 10 nM for melatonin and 300 μM for LA. Since melatonin has both direct free radical-scavenging actions and the ability to promote antioxidant enzyme activities, it seems it may be a highly beneficial molecule to protect skeletal muscle not only from toxin or drug exposure but also from the degenerative changes associated with aging. However, what percentage of its benefits derives from each of the multiple actions of the indoleamine remains to be investigated.

In summary, the current findings document that oxidative stress imposed on L6 myotubes causes atrophy and molecular damage, but also an attempt of a compensatory adaptive response which results in a weak expression of antioxidants that in turn may activate cellular defence mechanisms. Since a reduction in oxidative stress is an important target for drug-based therapies and strategies to prevent damage during aging-related diseases, discovering methods to attenuate it in all tissues including skeletal muscle could provide a means for protecting these vital cells from atrophy and aging-related pathophysiological processes. Further studies on this topic are mandatory in vitro, using also other additional skeletal muscle cell type, like C2C12 myotubes. C2C12 cells are grown as proliferative myoblasts and, upon reaching confluence, begin to differentiate, fusing into elongated, multinucleated and occasionally contractile fibres (Herbst et al. 2014), as L6 cells used in the present study. Moreover, to better assess the potential role of melatonin and LA as modulators of oxidative stress and skeletal muscle damage, other studies on their effect in combination will be an important and interesting issue for future study.
